# Toward an integrated view of nuclear pore transport

**DOI:** 10.1016/j.tcb.2026.04.002

**Published:** 2026-07

**Authors:** Luca Lanzano, Francesco Cardarelli

**Affiliations:** 1 Department of Physics and Astronomy “Ettore Majorana”, University of Catania, Catania, Italy; 2 NEST Laboratory, Scuola Normale Superiore, Piazza San Silvestro 12, Pisa, Italy

**Keywords:** nuclear pore complex, nucleocytoplasmic transport, biased transport, single-molecule tracking, super-resolution microscopy, fluorescence fluctuation analysis

## Abstract

The mechanism of molecular passage through nuclear pore complexes (NPCs) remains debated between passive diffusion and constrained transport. Integrating single-particle tracking, cargo-centric ensemble assays, and fluctuation analyses reveals that productive transport events are rapid, spatially confined, and biased, supporting a convergent phenomenology that provides an integrated view of NPC transport.

## Main text

Nuclear pore complexes (NPCs) are the sole gateways for molecular exchange between the nucleus and cytoplasm, a process fundamental to cellular homeostasis, gene regulation, and identity 1, 2. These assemblies must allow the rapid passage of thousands of cargo molecules per second while maintaining a robust barrier against unregulated traffic. Despite decades of investigation, the physical mechanism underlying this high selectivity and speed remains unresolved.

For many years, technical limitations hindered a molecular-level understanding of NPC transport. Early kinetic studies [3] and subsequent live-cell single-particle tracking [4] revealed heterogeneous behaviors and characteristic dwell times but lacked the spatial and temporal resolution required to resolve the fast, confined motion within the ~50-nm central channel.

One class of models framed NPC transport as facilitated diffusion. In this view, intrinsically disordered phenylalanine-glycine (FG)-repeat domains form a cohesive polymer meshwork that acts as an entropic sieve [5]. Inert macromolecules are excluded, whereas transport receptors transiently bind FG repeats and partition into the mesh, carrying their cargo across the barrier. This framework was refined in the selective phase model, in which FG domains form a distinct biophysical phase that selectively enriches transport receptors relative to the surrounding cytosol.

A second class of models emphasizes directional transport. Here, FG-nucleoporins are described as dynamic polymers whose interactions with transport receptors alter the effective energy landscape experienced by cargoes, as proposed in early reduction-of-dimensionality and polymer-brush-based models 6, 7. While initial support for receptor-induced FG reorganization came from planar *in vitro* systems, this conceptual dichotomy has since expanded into a broader landscape of mechanistic frameworks. These include virtual-gate, forest, gradient-based, and Kap-centric views, each highlighting different roles for FG-nucleoporin organization, transport-receptor occupancy, and pore geometry. Rather than attempting an exhaustive taxonomy, we refer the reader to a recent comparative review [8].

**Figure 1:**
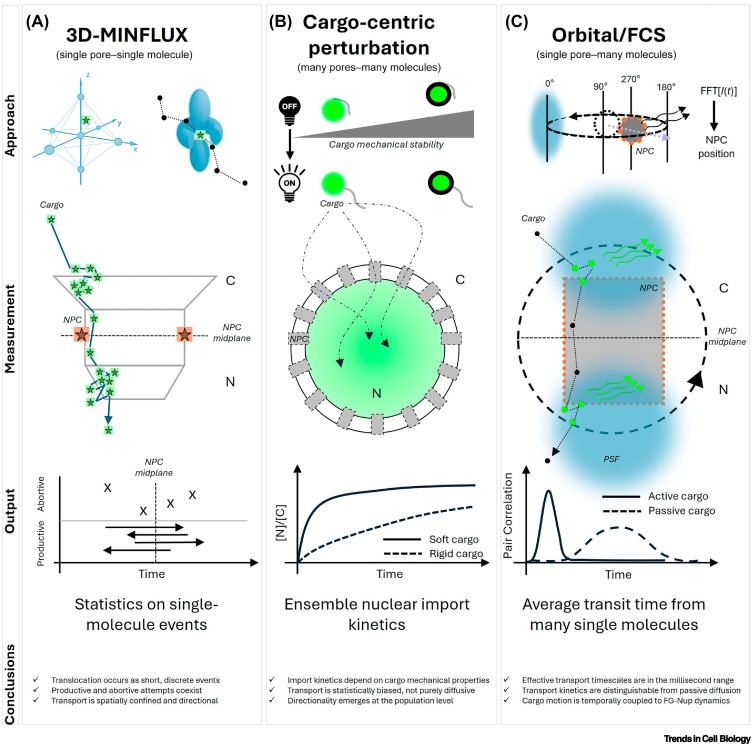
| Complementary experimental strategies to probe nuclear pore transport. (A) 3D minimal photon fluxes (MINFLUX) nanoscopy resolves trajectories of individual cargo molecules at single nuclear pore complexes (NPCs) with nanometer spatial precision and millisecond temporal resolution. This approach directly visualizes discrete translocation attempts, revealing short, spatially confined, and directional events, as well as the coexistence of productive and abortive crossings at the level of single pores and single molecules. Event-level outputs are represented schematically as productive translocation bursts crossing the NPC midplane and abortive attempts that fail to complete passage. (B) Cargo-centric ensemble perturbation assays probe nuclear import in live cells by monitoring fluorescence-based accumulation kinetics following controlled optogenetic resets. By systematically modifying cargo mechanical properties while preserving the import pathway, these assays quantify population-averaged nuclear import kinetics across many NPCs and many molecules, revealing statistically biased transport behavior that cannot be explained by passive diffusion alone and whose directionality emerges at the ensemble level. (C) Orbital tracking monitors the position of the point spread function (PSF) relative to the nuclear pore. Combined with fluorescence correlation spectroscopy (FCS) and pair-correlation function (pCF) analysis, processed via Fast Fourier Transform (FFT), this approach interrogates transport dynamics at single NPCs by analyzing ensemble-averaged molecular fluctuations generated by the passage of many individual cargo molecules. This approach extracts effective transport timescales in the millisecond range and reveals transport kinetics that are distinguishable from those expected for passive diffusion, as well as temporal coupling between cargo motion and phenylalanine-glycine (FG)-nucleoporin dynamics. Together, 3D-MINFLUX resolves single-molecule trajectories at individual nuclear pores; cargo-centric ensemble assays probe the collective response of many pores to many molecules; and fluctuation-based approaches interrogate transport at single pores through ensemble-averaged molecular fluctuations.

The landscape has been radically transformed by recent breakthroughs in super-resolution microscopy. In a landmark 2025 study, 3D minimal photon fluxes (MINFLUX) nanoscopy was used to track individual cargos traversing NPCs in digitonin-permeabilized cells, achieving nanometer spatial precision and millisecond temporal resolution [9]. This technical advance provided a detailed map of transport paths, revealing that import and export occur along overlapping annular (ring-shaped) trajectories that avoid the very center of the pore. A median ‘residence time’ of ~14 ms was reported for molecules within ± 25 nm of the NPC central plane. Although a major technical milestone, these measurements were performed in permeabilized cells, where cytoplasmic crowding and long-range constraints differ from intact cellular conditions. A closer inspection of the published trajectories and supplementary movies suggests that this residence time does not correspond to the translocation step alone. Productive crossings appear as short directional bursts of 2–4 localization steps (Figure 1A), corresponding to a few milliseconds. The longer ~14 ms residence time, therefore, likely reflects a combination of local exploration, binding, and productive passage through the pore.

This picture of rapid, biased motion is further supported by independent and complementary findings. A recent cargo-centric ensemble study showed anisotropic, direction-dependent nuclear import kinetics consistent with constrained progression across the NPC [10]. While this methodology does not resolve individual translocation events within the pore, it provides compelling evidence that transport is not well described by unrestricted Brownian diffusion (Figure 1B). Together, tracking and ensemble measurements indicate that once productive receptor–NPC interactions are established, nuclear transport is biased and dynamically constrained.

At the same time, both MINFLUX and earlier single-molecule tracking studies report frequent abortive NPC interactions alongside successful, highly directional translocation events. Recent residue-scale simulations provide a mechanistic framework consistent with this phenomenology, in which RanGTP-driven import proceeds via multivalent, hand-over-hand interactions between nuclear transport receptors and FG-rich nucleoporins along the surface of a dense FG-rich ring, with the nucleoporin Nsp1 guiding progression across the pore 11, 12. Together, these results support a view in which productive transport events follow biased, spatially confined pathways shaped by multivalent receptor–FG interactions and local barrier fluctuations, whereas many receptor–pore encounters remain abortive.

More than a decade before MINFLUX, our group developed an alternative strategy to probe fast NPC transport dynamics in intact cells. By combining orbital tracking of individual NPCs with fluorescence correlation spectroscopy (FCS) and fluorescence cross-correlation analysis, we extracted effective timescales of molecular transport without reconstructing individual trajectories 13, 14. In this framework, the NPC was treated as a functional ‘black box’, and transport dynamics were inferred from fluorescence fluctuations measured within a defined observation volume surrounding the pore (Figure 1C). Importantly, these effective timescales inherently reflect a convolution of multiple microscopic processes, including local exploration, binding interactions, and productive translocation across the NPC. Despite this intrinsic averaging, the extracted timescales were narrowly distributed and centered at ~3–5 ms [13], which is inconsistent with expectations for unrestricted diffusion and indicative of a kinetically well-defined transport process.

Dual-color cross-correlation measurements further revealed temporally correlated fluctuations between transiting cargos and the FG-nucleoporin Nup153, pointing to a tight temporal coupling between cargo dynamics and FG-Nup rearrangements during transport [14]. While these measurements do not provide direct spatial trajectories, their strength lies in the ability to probe transport dynamics in intact cells under physiological conditions. When viewed alongside MINFLUX measurements performed in permeabilized-cell systems, fluctuation-based approaches provide a complementary perspective on NPC transport. Although the measured timescales depend on methodological definitions and cellular context, both approaches converge on rapid, spatially confined, and biased transport events at the NPC.

The convergence of evidence from three independent and technically diverse experimental approaches—MINFLUX trajectory analysis [9], cargo-centric ensemble measurements [10], and FCS 13, 14—paints a coherent and compelling picture of NPC transport. The translocation of cargo across the central barrier is a rapid (millisecond-scale), spatially confined, and directional event, inconsistent with models of simple/random diffusion through a passive hydrogel.

This allows us to build a more unified mechanistic framework. In this view, FG-Nups act as dynamic, receptor-responsive polymers. The NPC is not a static gate but a ‘smart’ material that reconfigures itself in response to the right molecular key (the transport receptor). The binding of a receptor-cargo complex likely initiates a local, transient reorganization of FG-repeat interactions, enabling a low-energy pathway for translocation through rapid, multivalent exchange (‘hand-over-hand’) under strong steric constraints. Importantly, while receptor-induced collapse was supported by early planar FG-brush experiments 6, 7, recent 3D NPC-mimetic measurements and residue-scale simulations suggest that high receptor partitioning can be accommodated with only modest changes in the global FG-mesh conformation 11, 12, implying that directionality does not require large-scale FG collapse.

This emerging consensus helps redefine the most pressing experimental questions for the future. The challenge is no longer to ask ‘if’ transport is directional, but to understand ‘how’ this directionality is generated and regulated. Future studies will need to move toward multicolor, high-speed tracking experiments capable of simultaneously visualizing cargos, their transport receptors, and selected FG-Nups in real-time. Such experiments could directly capture transient FG–receptor interactions, local rearrangements of the FG meshwork, and hand-over-hand transfer events during productive transport. An additional key question is how these local, dynamic processes are coupled to the cell’s broader regulatory systems, such as the RanGTP gradient that ultimately enforces the directionality of import and export. Integrating high-resolution tracking with functional readouts of the Ran system will be essential to link molecular-scale dynamics to cellular-scale transport regulation.

More than a decade ago, statistical analysis suggested that NPC transport was a fast, directional, and FG-mediated process 13, 14. Today, both direct visualization [9] and ensemble measurements [10] confirm this view. Far from being contradictory, these different methodologies have proven to be deeply complementary, providing a powerful example of how integrating insights from orthogonal approaches can address long-standing biological puzzles. The NPC is not a passive sieve; it is a sophisticated and responsive molecular machine. The exciting task ahead lies in dissecting its beautiful mechanics in even greater detail.
